# A Secure and Lightweight Authentication Protocol for IoT-Based Smart Homes

**DOI:** 10.3390/s21041488

**Published:** 2021-02-21

**Authors:** JiHyeon Oh, SungJin Yu, JoonYoung Lee, SeungHwan Son, MyeongHyun Kim, YoungHo Park

**Affiliations:** 1 School of Electronic and Electrical Engineering, Kyungpook National University, Daegu 41566, Korea; chldlstnr071@knu.ac.kr (J.O.); darkskiln@knu.ac.kr (S.Y.); harry250@knu.ac.kr (J.L.); sonshawn@knu.ac.kr (S.S.); kimmyeong123@knu.ac.kr (M.K.); 2 Electronics and Telecommunications Research Institute, Daejeon 34129, Korea; 3 School of Electronics Engineering, Kyungpook National University, Daegu 41566, Korea

**Keywords:** smart homes, IoT, authentication, BAN logic, ROR model, AVISPA

## Abstract

With the information and communication technologies (ICT) and Internet of Things (IoT) gradually advancing, smart homes have been able to provide home services to users. The user can enjoy a high level of comfort and improve his quality of life by using home services provided by smart devices. However, the smart home has security and privacy problems, since the user and smart devices communicate through an insecure channel. Therefore, a secure authentication protocol should be established between the user and smart devices. In 2020, Xiang and Zheng presented a situation-aware protocol for device authentication in smart grid-enabled smart home environments. However, we demonstrate that their protocol can suffer from stolen smart device, impersonation, and session key disclosure attacks and fails to provide secure mutual authentication. Therefore, we propose a secure and lightweight authentication protocol for IoT-based smart homes to resolve the security flaws of Xiang and Zheng’s protocol. We proved the security of the proposed protocol by performing informal and formal security analyses, using the real or random (ROR) model, Burrows–Abadi–Needham (BAN) logic, and the Automated Validation of Internet Security Protocols and Applications (AVISPA) tool. Moreover, we provide a comparison of performance and security properties between the proposed protocol and related existing protocols. We demonstrate that the proposed protocol ensures better security and lower computational costs than related protocols, and is suitable for practical IoT-based smart home environments.

## 1. Introduction

With the development of information and communication technologies (ICT) and Internet of Things (IoT), smart home automation systems are receiving a lot of attention. The smart home is a networking environment that connects smart devices (e.g., IoT and sensors) to each other. Based on these smart devices, users can utilize various home services. When the user is inside the home, the user can control all smart devices with a voice commands or applications, granting the user accesses to services such as turning the TV on/off, choosing music, switching lights on/off, and so on. When the user is outside the home, the user can monitor and control various smart devices by checking their status. Thus, users can enjoy a high level of comfort and an increased quality of life through smart home environments.

Generally, smart home environments consist of the user, smart devices, a home gateway, and a registration authority [1,2,3]. A remote user wants to use the data collected by smart devices. However, smart devices are resource limited in terms of computational power, amount of memory, and bandwidth [4]. For these reasons, smart devices communicate through the home gateway. The home gateway acts as a bridge between smart devices and remote users by providing short and long-distance wireless communication interfaces that maintain the connectivity with internal smart devices and remote users [5]. Users can remotely operate smart devices with the help of a home gateway using Internet-enabled mobile phones and tablets anytime and anywhere. Thus, the home gateway plays a crucial role by controlling the data exchange. It manages the communication between internal and external surroundings.

Unfortunately, the smart home has security and privacy problems because the sensitive data collected by smart devices are exchanged through wireless networks. If an adversary obtains the data, the adversary will abuse them for his own purposes. Thus, security and privacy are essential elements to providing secure home services. In addition, the exchanged data should meet confidentiality, integrity, and availability standards. Asymmetric and symmetric key cryptosystems are inappropriate for applying to low-capacity devices because they generate high computational costs. Thus, secure and lightweight authentication protocols are necessary to provide security and privacy in IoT-based smart homes.

In 2020, Xiang and Zheng [6] proposed a situation-aware protocol for device authentication in smart grid-enabled smart home environments. Xiang and Zheng claimed that their protocol can withstand impersonation, man-in-the-middle (MITM), and replay attacks. Xiang and Zheng also demonstrated that their protocol can provide data integrity and mutual authentication. However, herein we prove that their protocol does not prevent stolen smart device, impersonation, and session key disclosure attacks, and fails to ensure mutual authentication. They also mentioned that their protocol concentrates on the security of smart grid-enabled smart home environments. However, they proposed an authentication protocol that is only for smart home environments. Thus, we focus on general smart home environments and present a secure and lightweight authentication protocol for IoT-based smart homes that deals with the security drawbacks of Xiang and Zheng’s protocol [6]. The proposed protocol is efficient for resource-constrained smart devices because we use only one-way hash functions and XOR operations.

### 1.1. Contributions

This paper has the following main contributions.

- We analyze the security vulnerabilities of Xiang and Zheng’s protocol [6]. To resolve the security drawbacks of their protocol, we propose a secure and lightweight authentication protocol for IoT-based smart homes.
- We demonstrate that our protocol is secure against various kinds of known attacks by reporting on an informal security analysis.
- We conducted formal analysis using the Automated Validation of Internet Security Protocols and Applications (AVISPA) tool [7,8,9], Burrows–Abadi–Needham (BAN) logic [10], and the real or random (ROR) model [11]. With the formal analysis, we proved secure mutual authentication, the session key security, and the resistance against MITM and replay attacks of our protocol.
- We provide a comparison of performance and security properties between our protocol and related protocols. The results show that our protocol provides better security and computational costs compared to related protocols.

### 1.2. Adversary Model

We adopted the widely-used Dolev–Yao (DY) threat model [12,13,14] and the Canetti and Krawczyk (CK) adversary threat model [15,16] to evaluate the security of the proposed protocol. The capabilities of an adversary A can be defined as follows.

- A can eavesdrop, intercept, inject, replay, and modify transmitted messages via a public channel and then A can perform MITM, replay, impersonation attacks, etc. [17].
- A can steal the legal user’s mobile device or smart device and extract secret credentials stored in the memory by performing the power analysis attack [18,19,20,21].
- A can access short-term keys, long-term keys, and session states of each party.

In addition, we developed some assumptions for our protocol. A cannot feasibly guess the identity and password of the mobile user simultaneously [22,23,24]. A cannot extract the data stored in the home gateway’s database, since the home gateway has a secure database.

### 1.3. Organization

The remaining parts of this paper are structured as follows. In Section 2, we briefly discuss existing proposed protocols in IoT-based smart homes. We suggest the system model of the proposed protocol in Section 3. We review Xiang and Zheng’s protocol in Section 4 and analyze security weaknesses of Xiang and Zheng’s protocol in Section 5. Section 6 proposes a secure and lightweight authentication protocol for IoT-based smart homes to improve the security drawbacks of Xiang and Zheng’s protocol. Section 7 analyzes the security of our protocol through informal and formal analyses with BAN logic, the ROR model, and the AVISPA tool. In Section 8, we present the results of performance and security property comparisons between the proposed protocol and related protocols. Finally, we present the conclusion in Section 9.

## 2. Related Works

In the last few years, many researchers proposed authentication protocols to provide secure communication between users and smart devices in smart home environments. Santoso and Vun [25] proposed a secure authentication protocol using elliptic curve cryptography (ECC) in IoT-based smart homes. Several authors [26,27] revealed that Santoso and Vun’s protocol [25] is vulnerable to privileged-insider and stolen smart card attacks, and fails to achieve user anonymity and untraceability. Dey and Hossian [28] presented a secure session key establishment protocol for smart home environments using public key cryptosystems. Dey and Hossian [28] proved that their protocol achieves resilience against various attacks. Unfortunately, some researchers [29,30] pointed out that Dey and Hossian’s protocol [28] has various security drawbacks, such as device compromised and known-key attacks, and is unsuccessful in ensuring anonymity and confidentiality. Shuai et al. [31] suggested an ECC-based anonymous authentication protocol for smart home environments. These protocols [25,28,31] use asymmetric key cryptosystems such as ECC for smart home security. However, in terms of costs, symmetric key cryptosystems are more efficient than asymmetric key cryptosystems for deployment on resource-constrained smart devices.

In view of the computational cost for low capacity devices, many authentication protocols have been proposed using symmetric key cryptosystems in smart home environments. Vaidya et al. [32] proposed a robust authentication protocol to provide secure remote access in home environments using symmetric key cryptosystems. Vaidya et al. [32] claimed that their protocol resists synchronization and stolen smart card attacks, and provides forward secrecy and mutual authentication. However, Kim and Kim [33] demonstrated that Vaidya et al.’s protocol [32] does not resist password guessing and smart card loss attacks, and does not provide forward secrecy. To resolve the security problems in Vaidya et al.’s protocol [32], Kim and Kim [33] proposed an improved authentication protocol. Wazid et al. [34] proposed a symmetric key-based secure remote user authentication protocol to provide future secure communications. Wazid et al. [34] proved that their protocol is secure against other possible known attacks. Lyu et al. [35] pointed out that Wazid et al.’s protocol [34] is not secure against desynchronization and compromised server attacks. Poh et al. [36] proposed a privacy-preserving authentication protocol to support data confidentiality. Unfortunately, Irshad et al. [37] pointed out that Poh et al.’s protocol [36] cannot maintain the privacy of authentication parameters. Although these protocols [32,33,34,35,36] use symmetric key cryptosystems considering the low capacity devices, symmetric key cryptosystems are still unacceptable for smart devices with limited resources in terms of computational costs.

Recently, several lightweight authentication protocols [6,38] have been proposed for smart home environments to solve these problems. Banerjee et al. [38] presented an anonymous and robust authentication protocol for IoT-based smart homes using one-way hash functions, XOR operations, and a fuzzy extractor. Banerjee et al. [38] proved that their protocol resists various attacks. However, AL-Turjman and Deebak [39] pointed out that Banerjee et al.’s protocol [38] does not provide identity protection, traceability, or session secret key agreement. Xiang and Zheng [6] presented a situation-aware protocol for device authentication in smart home environments. Xiang and Zheng [6] claimed that their protocol resists various security threats and ensures data integrity and mutual authentication. However, we prove here that Xiang and Zheng’s protocol [6] cannot ensure secure mutual authentication and is vulnerable to stolen smart device, impersonation, and session key disclosure attacks. Therefore, we propose a secure and lightweight authentication protocol for IoT-based smart homes to improve the security flaws of Xiang and Zheng’s protocol [6].

## 3. System Model

**Figure 1:**
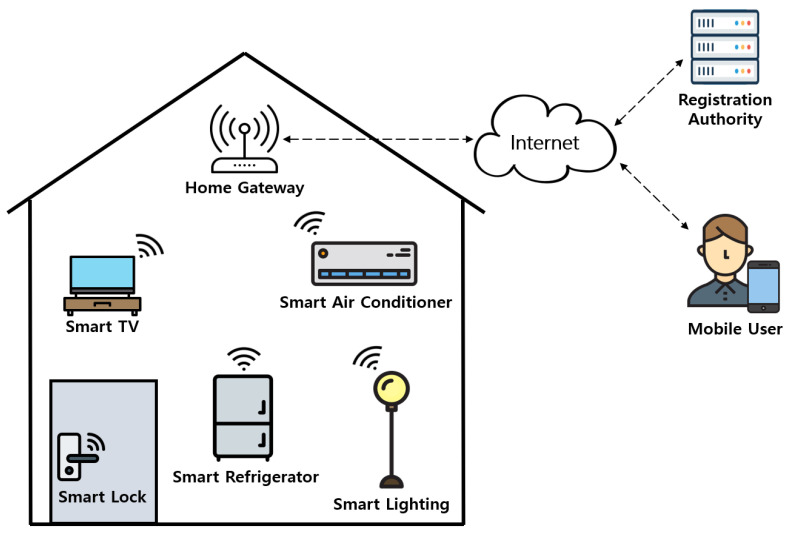
System model for IoT-based smart homes.

Xiang and Zheng [6] claimed that their protocol concentrates on the security of smart grid-enabled smart home environments, but they proposed an authentication protocol that is only for smart home environments. Therefore, we focus on the architecture of general IoT-based smart home environments. The system model is shown in Figure 1.

The proposed system is composed of a mobile user (MU), a smart device (SD), a home gateway (HGW), and a registration authority (RA). RA and HGW are trusted entities in smart home environments. RA is responsible for initializing the system and registering MU and SD. MU first needs to register at RA to utilize services. SD and HGW also need to register at RA for providing home services. After receiving the registration request message from MU and SD, RA stores the information of each entity in the mobile device of MU and in the memory of SD. RA also stores all information required for the authentication of the MU and SD in HGW’s database. Then, the MU and SD perform the mutual authentication and session key agreement with the help of the HGW. With this session key, MU and SD can utilize secure smart home services.

## 4. Review of Xiang and Zheng’s Protocol

This section reviews Xiang and Zheng’s protocol [6]. Xiang and Zheng proposed an authentication protocol according to the security risk level in smart home environments. Their protocol consists of smart device registration, and authentication and key agreement phases. The notation of this paper is described in Table 1.

### 4.1. Smart Device Registration Phase

At the registration phase, RA generates an identity $ID_{SD}$ and a random number $r_{RA}$ for SD and computes $S_{i}=h(ID_{SD}\left|\right|r_{RA})$. Then, RA sends $\left\{ID_{SD},S_{i}\right\}$ to SD and $\left\{ID_{SD},r_{RA}\right\}$ to HGW through a secure channel.

### 4.2. Authentication and Key Agreement Phase

After the registration, SD sends the message $MSG_{1}=[HE_{1}||ID_{SD}]$ to HGW in the authentication and key agreement phase. $HE_{1}{=}^{\prime }SD-AUTH^{\prime }$ is a message header of $MSG_{1}$. Upon getting $MSG_{1}$, HGW receives the current situation from the smart home system regarding whether the security risk level is low or high. According to the security risk level, the authentication phase is divided into low security risk and high security risk.

#### 4.2.1. Low Security Risk

When HGW receives a low-security-risk level report, the authentication phase is described below.

**Step 1:**  HGW computes $S_{i}^{*}=h(ID_{SD}^{*}\left|\right|r_{RA})$ and extracts current timestamp $T_{1}$. Then HGW calculates $C_{1,L}=(ID_{G}\left|\right|T_{1})⊕S_{i}^{*}$ and $C_{2,L}=h(HE_{2,L}||ID_{G}\left|\right|T_{1}\left|\right|S_{i}^{*})$. Finally, HGW sends $MSG_{2,L}=[HE_{2,L}\left|\right|C_{1,L}\left|\right|C_{2,L}]$ to SD, where $HE_{2,L}{=}^{\prime }HGW-LOW^{\prime }$ is the header of the message $MSG_{2,L}$ through an insecure channel.
**Step 2:**  Upon receiving the message $MSG_{2,L}$ at timestamp $T_{1}^{\prime }$, SD knows the current security risk level is low from the message header. SD also computes $C_{2,L}^{*}=h(HE_{2,L}^{*}||ID_{G}^{*}\left|\right|T_{1}^{*}\left|\right|S_{i})$ and checks if $|T_{1}^{\prime }-T_{1}^{*}|\leq \Delta T$ and $C_{2,L}^{*}\overset{?}{=}C_{2,L}$. If it is not equal, the authentication process will be aborted. Then, SD computes $A_{i}=h(ID_{G}^{*}||h(ID_{SD}\left|\right|$$S_{i}))$ and extracts the current timestamp $T_{2}$. SD also computes $C_{3,L}=(ID_{SD}\left|\right|T_{2})⊕A_{i}$ and $C_{4,L}=h(HE_{3,L}||ID_{SD}\left|\right|T_{2}\left|\right|A_{i})$. Finally, SD sends $MSG_{3,L}=[HE_{3,L}\left|\right|C_{3,L}\left|\right|C_{4,L}]$ to HGW, where $HE_{3,L}{=}^{\prime }SD-LOW^{\prime }$ is the header of the message $MSG_{3,L}$. SD computes the session key $SK=h(T_{1}^{*}||T_{2}||S_{i}||A_{i})$ for the future data communication.
**Step 3:**  After receiving $MSG_{3,H}$ at timestamp $T_{2}^{\prime }$, HGW computes $A_{i}^{*}=h(ID_{G}||h(ID_{SD}\left|\right|S_{i}^{*}))$, $(ID_{SD}^{*}\left|\right|T_{2}^{*})=C_{3,L}⊕A_{i}^{*}$, and $C_{4,L}^{*}=h(HE_{3,L}^{*}||ID_{SD}^{*}\left|\right|T_{2}^{*}\left|\right|A_{i}^{*})$. Then, HGW checks if $|T_{2}^{\prime }-T_{2}^{*}|\leq \Delta T$ and $C_{4,H}^{*}\overset{?}{=}C_{4,H}$. If it is correct, HGW computes the session key $SK=h(T_{1}||T_{2}^{*}||S_{i}^{*}||A_{i}^{*})$ and adds $ID_{SD}$ to the trusted device list.

#### 4.2.2. High Security Risk

If HGW receives a situation report detailing that the current security risk level is high, the authentication phase contains the following steps.

**Step 1:**  HGW computes $S_{i}^{*}=h(ID_{SD}^{*}\left|\right|r_{RA})$, and generates a random number $RN_{G}$. After that, HGW extracts a current timestamp $T_{1}$, and computes $C_{1,H}=E_{S_{i}^{*}}(ID_{G}\left|\right|T_{1}||RN_{G})$ and $C_{2,H}=h(HE_{2,H}||ID_{G}\left|\right|T_{1}||RN_{G})$. Then, HGW sends the message $MSG_{2,H}=[HE_{2,H}\left|\right|C_{1,H}\left|\right|C_{2,H}]$ to SD, where $HE_{2,H}{=}^{\prime }HGW-HIGH^{\prime }$ is the message header of $MSG_{2,H}$ through a public channel.
**Step 2:**  After getting $MSG_{2,H}$ at timestamp $T_{1}^{\prime }$, SD knows the security risk level is high from the header of $MSG_{2,H}$. SD then computes $(ID_{G}^{*}\left|\right|T_{1}^{*}||RN_{G}^{*})=D_{S_{i}}\left(C_{1,H}^{*}\right)$ and $C_{2,H}=h(HE_{2,H}^{*}||ID_{G}^{*}\left|\right|T_{1}^{*}||RN_{G}^{*})$. After that, SD checks whether $|T_{1}^{\prime }-T_{1}^{*}|\leq \Delta T$ and $C_{2,H}^{*}\overset{?}{=}C_{2,H}$. If the check is failed, the authentication process will be terminated. Otherwise, SD computes $A_{i}=h(ID_{G}^{*}||h(ID_{SD}\left|\right|S_{i}))$ and generates a random number $RN_{SD}$. Then, SD extracts the current timestamp $T_{2}$, and computes $C_{3,H}=E_{A_{i}}(ID_{SD}\left|\right|T_{2}||RN_{SD})$ and $C_{4,H}=h(HE_{3,H}||ID_{SD}\left|\right|T_{2}||RN_{SD})$. Finally, SD sends the message $MSG_{3,H}=[HE_{3,H}\left|\right|C_{3,H}\left|\right|C_{4,H}]$ to HGW, where $HE_{3,H}{=}^{\prime }SD-HIGH^{\prime }$ is the message header of $MSG_{3,H}$, and computes the session key $SK=h(T_{1}^{*}||T_{2}||S_{i}||A_{i}||$$RN_{SD}||RN_{G}^{*})$.
**Step 3:**  Upon receiving $MSG_{3,H}$ at timestamp $T_{2}^{\prime }$, HGW computes $A_{i}^{*}=h(ID_{G}||h(ID_{SD}\left|\right|S_{i}^{*}))$, $(ID_{SD}^{*}\left|\right|T_{2}^{*}||RN_{SD}^{*})=D_{A_{i}^{*}}\left(C_{3,H}\right)$, and $C_{4,H}^{*}=h(HE_{3,H}^{*}||ID_{SD}^{*}\left|\right|T_{2}^{*}||RN_{SD}^{*})$. Then, HGW checks whether $|T_{2}^{\prime }-T_{2}^{*}|\leq \Delta T$ and $C_{4,H}^{*}\overset{?}{=}C_{4,H}$. If it is correct, HGW computes the session key $SK=h(T_{1}||T_{2}^{*}||S_{i}^{*}||A_{i}^{*}||RN_{SD}^{*}||RN_{G})$ and adds $ID_{SD}$ to the trusted device list.

## 5. Cryptanalysis of Xiang and Zheng’s Protocol

In this section, we discuss the security flaws of Xiang and Zheng’s protocol. We demonstrate that their protocol is vulnerable to various attacks and does not perform secure mutual authentication.

### 5.1. Stolen Smart Device Attack

We suppose that an adversary A can obtain secret credentials $\left\{ID_{SD},S_{i}\right\}$ of SD using the power analysis according to Section 1.2. Xiang and Zheng’s protocol sends the authentication request message $MSG_{1}=[HE_{1}||ID_{SD}]$ as plaintext. A can obtain $HE_{1}$ from $\left[HE_{1}||ID_{SD}\right]$ of the previous session. Then, A can make the message $MSG_{1}$ anytime and perform various attacks with secret credentials. In conclusion, their protocol does not prevent the stolen smart device attack.

### 5.2. Impersonation Attack

According to Section 1.2, A can perform an impersonation attack at low and low-security-risk levels. The detailed processes are below.

#### 5.2.1. Low Security Risk

A can perform the impersonation attack with the following steps.

**Step 1:**  With the obtained secret credentials $\left\{ID_{SD},S_{i}\right\}$ from SD and $HE_{1}$ from the previous session, A can send the message $MSG_{1}=[HE_{1}||ID_{SD}]$.
**Step 2:**  Upon getting $MSG_{1}$, HGW computes $S_{i}^{*}=h(ID_{SD}^{*}\left|\right|r_{RA})$ and extracts the current timestamp $T_{1}$. After that, HGW computes $C_{1,L}=(ID_{G}\left|\right|T_{1})⊕S_{i}^{*}$ and $C_{2,L}=h(HE_{2,L}||ID_{G}\left|\right|T_{1}\left|\right|S_{i}^{*})$, and sends the message $MSG_{2,L}=[HE_{2,L}\left|\right|C_{1,L}\left|\right|C_{2,L}]$.
**Step 3:**  After receiving $MSG_{2,L}$, A computes $(ID_{G}^{*}\left|\right|T_{1}^{*})=C_{1,L}⊕S_{i}$ and $C_{2,L}^{*}=h(HE_{2,L}^{*}||ID_{G}^{*}\left|\right|T_{1}^{*}\left|\right|S_{i})$. Then, A verifies the validity of $T_{1}^{*}$ and $C_{2,L}^{*}$. If it is equal, A computes $A_{i}=h(ID_{G}^{*}||h(ID_{SD}\left|\right|S_{i}))$ and generates the current timestamp $T_{2}$. After that, A computes $C_{3,L}=(ID_{SD}\left|\right|T_{2})⊕A_{i}$ and $C_{4,L}=h(HE_{3,L}||ID_{SD}\left|\right|T_{2}\left|\right|A_{i})$. Finally, A sends the message $MSG_{3,L}=[HE_{3,L}\left|\right|C_{3,L}\left|\right|C_{4,L}]$ to HGW and computes the session key $SK=h(T_{1}^{*}||T_{2}||S_{i}||A_{i})$.
**Step 4:**  Upon getting $MSG_{3,L}$, HGW computes $A_{i}^{*}=h(ID_{G}||h(ID_{SD}\left|\right|S_{i}^{*}))$, $(ID_{SD}^{*}\left|\right|T_{2}^{*})=C_{3,L}⊕A_{i}^{*}$, and $C_{4,L}^{*}=h(HE_{3,L}^{*}||ID_{SD}^{*}\left|\right|T_{2}^{*}\left|\right|A_{i}^{*})$. After that, HGW checks the validity of $T_{2}^{*}$ and $C_{4,L}^{*}$. If it is equal, HGW computes $SK=h(T_{1}||T_{2}^{*}||S_{i}^{*}||A_{i}^{*})$.

Thus, A can impersonate SD successfully, and Xiang and Zheng’s protocol cannot prevent the impersonation attack at the low-security-risk level.

#### 5.2.2. High Security Risk

With the obtained secret credentials $\left\{ID_{SD},S_{i}\right\}$, A can disguise as SD, and the detailed steps are below.

**Step 1:**  A can send $MSG_{1}=[HE_{1}||ID_{SD}]$ to HGW using obtained secret credentials $\left\{ID_{SD},S_{i}\right\}$ and $HE_{1}$.
**Step 2:**  Upon getting $MSG_{1}$, HGW calculates $S_{i}^{*}=h(ID_{SD}^{*}\left|\right|r_{RA})$ and generates a random number $RN_{G}$. After that, HGW extracts the current timestamp $T_{1}$, and computes $C_{1,H}=E_{S_{i}^{*}}(ID_{G}\left|\right|T_{1}||RN_{G})$ and $C_{2,H}=h(HE_{2,H}||ID_{G}\left|\right|T_{1}||RN_{G})$. Then, HGW sends $MSG_{2,H}=[HE_{2,H}\left|\right|C_{1,H}\left|\right|C_{2,H}]$.
**Step 3:**  After receiving $MSG_{2,H}$, A computes $(ID_{G}^{*}\left|\right|T_{1}^{*}||RN_{G})=D_{S_{i}}\left(C_{1,H}^{*}\right)$ and $C_{2,H}^{*}=h(HE_{2,H}^{*}||ID_{G}^{*}\left|\right|T_{1}^{*}||RN_{G}^{*})$. Then, A verifies the validity of $T_{1}^{*}$ and $C_{2,H}^{*}$. If all checks pass, A computes $A_{i}^{*}=h(ID_{G}^{*}||h(ID_{SD}\left|\right|S_{i}))$, generates a random number $RN_{SD}$, and extracts the current timestamp $T_{2}$. After that, A computes $C_{3,H}=E_{A_{i}}(ID_{SD}\left|\right|T_{2}\left|\right|$$RN_{SD})$, $C_{4,H}=h(HE_{3,H}||ID_{SD}\left|\right|T_{2}||RN_{SD})$, and $SK=h(T_{1}^{*}||T_{2}||S_{i}||A_{i}||RN_{SD}||RN_{G}^{*})$. Finally, A sends $MSG_{3,H}=[HE_{3,H}\left|\right|C_{3,H}\left|\right|C_{4,H}]$ to HGW.
**Step 4:**  Upon getting $MSG_{3,H}$, HGW computes $A_{i}^{*}=h(ID_{G}||h(ID_{SD}\left|\right|S_{i}^{*}))$, $(ID_{SD}^{*}\left|\right|T_{2}^{*}||RN_{SD}^{*})=D_{A_{i}^{*}}\left(C_{3,H}\right)$, and $C_{4,H}^{*}=h(HE_{3,H}^{*}||ID_{SD}^{*}\left|\right|T_{2}^{*}||RN_{SD}^{*})$. Then, HGW checks the validity of $T_{2}^{*}$ and $C_{4,H}^{*}$. If it is equal, HGW computes $SK=h(T_{1}||T_{2}^{*}||S_{i}^{*}||A_{i}^{*}||RN_{SD}^{*}||RN_{G})$.

In conclusion, Xiang and Zheng’s protocol cannot prevent the impersonation attack at the low-security-risk level because A can impersonate SD successfully.

### 5.3. Session Key Disclosure Attack

As mentioned in Section 1.2, A can extract secret credentials $\left\{ID_{SD},S_{i}\right\}$. In addition, according to Section 5.2, A can obtain the session key between SD and HGW at the both low-security-risk and high-security-risk levels. With the obtained session key, A can communicate with HGW and misinform HGW for A’s own purpose. Therefore, Xiang and Zheng’s protocol is vulnerable to the session key disclosure attack.

### 5.4. Mutual Authentication

Xiang and Zheng claimed that their protocol supports the mutual authentication between SD and HGW because $S_{i}$ and $A_{i}$ cannot be obtained from the eavesdropped messages. However, in accordance with Section 5.2, A can generate an authentication request message $MSG_{1}=[HE_{1}||ID_{SD}]$ and calculate session key $SK=h(T_{1}||T_{2}||S_{i}||A_{i})$ and $SK=h(T_{1}||T_{2}||S_{i}||A_{i}||RN_{SD}||RN_{G})$ at low security and low security phases, respectively. Thus, Xiang and Zheng’s protocol does not satisfy secure mutual authentication between SD and HGW.

## 6. Proposed Protocol

In this section, we present a secure and lightweight authentication protocol for IoT-based smart homes to improve the security drawbacks of Xiang and Zheng’s protocol [6]. The proposed protocol consists of four phases: initialization, registration, authentication and key agreement, and password update.

### 6.1. Initialization Phase

Before SD and HGW are deployed in the smart home, RA generates a master key $K_{RA}$. HGW has a unique identity $ID_{G}$, and SD has a unique identity $ID_{SD}$ and secret key $K_{SD}$.

### 6.2. Registration Phase

The detailed registration phases for the smart device and user are below.

#### 6.2.1. Smart Device Registration Phase

**Figure 2:**
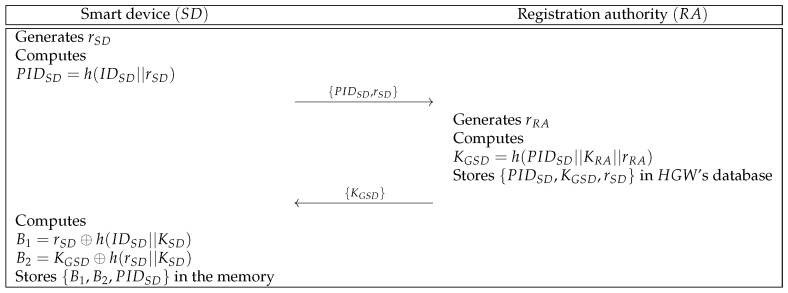
Smart device registration phase of the proposed protocol.

To provide home services to MU, SD must register at RA. We indicate the registration phase of SD and RA in Figure 2, and detailed steps are described below.

**Step 1:**  SD generates a random number $r_{SD}$ and computes $PID_{SD}=h(ID_{SD}\left|\right|r_{SD})$. Then, SD sends $\left\{PID_{SD},r_{SD}\right\}$ to RA through a secure channel.
**Step 2:**  Upon getting the message, RA generates $r_{RA}$ and computes $K_{GSD}=h(PID_{SD}\left|\right|K_{RA}\left|\right|r_{RA})$. Then, RA stores $\left\{PID_{SD},K_{GSD},r_{SD}\right\}$ in HGW’s database and sends $\left\{K_{GSD}\right\}$ to SD over a secure channel. After that, RA makes $PID_{SD}$ public.
**Step 3:**  After receiving the message, SD computes $B_{1}=r_{SD}⊕h(ID_{SD}\left|\right|K_{SD})$ and $B_{2}=K_{GSD}⊕h(r_{SD}\left|\right|K_{SD})$. Then, SD stores $\left\{B_{1},B_{2},PID_{SD}\right\}$ in the memory.

#### 6.2.2. Mobile User Registration Phase

**Figure 3:**
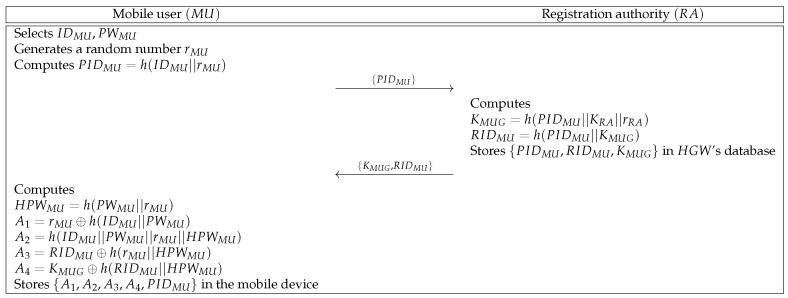
Mobile user registration phase of the proposed protocol.

MU must register at RA to use the data transmitted from SD. Figure 3 shows the registration phase of MU and RA. This phase is described as follows.

**Step 1:**  MU selects identity and password $\left\{ID_{MU},PW_{MU}\right\}$ and generates a random number $r_{MU}$. Then, MU computes $PID_{MU}=h(ID_{MU}\left|\right|r_{MU})$ and sends $\left\{PID_{MU}\right\}$ to RA through a secure channel.
**Step 2:**  Upon receiving the message, RA computes $K_{MUG}=h(PID_{MU}\left|\right|K_{RA}\left|\right|r_{RA})$ and $RID_{MU}=h(PID_{MU}\left|\right|K_{MUG})$. Then, RA stores $\left\{PID_{MU},RID_{MU},K_{MUG}\right\}$ in HGW’s database and sends $\left\{K_{MUG},RID_{MU}\right\}$ to MU via a secure channel.
**Step 3:**  After receiving the message, MU computes $HPW_{MU}=h(PW_{MU}\left|\right|r_{MU})$, $A_{1}=r_{MU}⊕h(ID_{MU}||PW_{MU})$, $A_{2}=h(ID_{MU}||PW_{MU}\left|\right|r_{MU}||HPW_{MU})$, $A_{3}=RID_{MU}⊕h(r_{MU}||HPW_{MU})$, and $A_{4}=K_{MUG}⊕h(RID_{MU}||HPW_{MU})$. Then, MU stores $\left\{A_{1},A_{2},A_{3},A_{4},PID_{MU}\right\}$ in the mobile device.

### 6.3. Authentication and Key Agreement Phase

**Figure 4:**
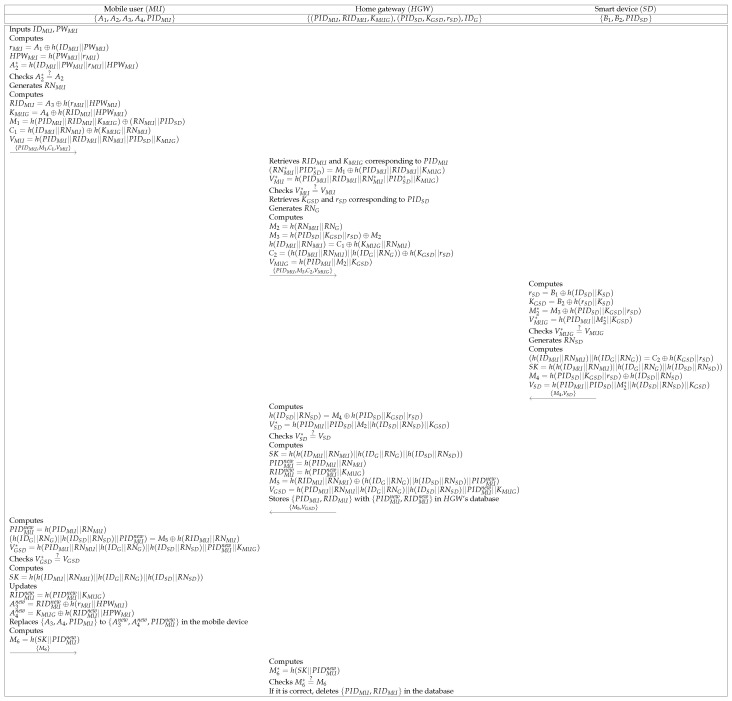
Authentication and key agreement phase of the proposed protocol.

To utilize secure home services, MU and SD establish a session key with the help of HGW. We indicate the detailed steps below, and a summarized version of this phase is in Figure 4.

**Step 1:**  MU inputs identity and password $\left\{ID_{MU},PW_{MU}\right\}$ and computes $r_{MU}=A_{1}⊕h(ID_{MU}||PW_{MU})$, $HPW_{MU}=h(PW_{MU}\left|\right|r_{MU})$, and $A_{2}^{*}=h(ID_{MU}||PW_{MU}\left|\right|r_{MU}||HPW_{MU})$. Then, MU checks if $A_{2}^{*}\overset{?}{=}A_{2}$. If this condition is satisfied, MU generates a random nonce $RN_{MU}$ and computes $RID_{MU}=A_{3}⊕h(r_{MU}||HPW_{MU})$, $K_{MUG}=A_{4}⊕h(RID_{MU}||HPW_{MU})$, $M_{1}=h(PID_{MU}||RID_{MU}\left|\right|K_{MUG})⊕(RN_{MU}||PID_{SD})$, $C_{1}=h(ID_{MU}||RN_{MU})⊕h(K_{MUG}||RN_{MU})$, and $V_{MU}=h(PID_{MU}||RID_{MU}||RN_{MU}||PID_{SD}\left|\right|K_{MUG})$. After that, MU sends $\left\{PID_{MU},M_{1},C_{1},V_{MU}\right\}$ to HGW through a public channel.
**Step 2:**  Upon getting the message, HGW retrieves $RID_{MU}$ and $K_{MUG}$ corresponding to $PID_{MU}$, and computes $(RN_{MU}^{*}||PID_{SD}^{*})=M_{1}⊕h(PID_{MU}||RID_{MU}\left|\right|K_{MUG})$ and $V_{MU}^{*}=h(PID_{MU}||RID_{MU}||RN_{MU}^{*}||PID_{SD}^{*}\left|\right|K_{MUG})$. HGW checks if $V_{MU}^{*}\overset{?}{=}V_{MU}$. If it is equal, HGW retrieves $K_{GSD}$ and $r_{SD}$ corresponding to $PID_{SD}$. Then, HGW generates a random nonce $RN_{G}$ and computes $M_{2}=h(RN_{MU}||RN_{G})$, $M_{3}=h(PID_{SD}\left|\right|$$K_{GSD}\left|\right|r_{SD})⊕M_{2}$, $h(ID_{MU}||RN_{MU})=C_{1}⊕h(K_{MUG}||RN_{MU})$, $C_{2}=(h(ID_{MU}\left|\right|$$RN_{MU})||h(ID_{G}||RN_{G}))⊕h(K_{GSD}\left|\right|r_{SD})$, and $V_{MUG}=h(PID_{MU}\left|\right|M_{2}\left|\right|K_{GSD})$. Finally, HGW sends $\left\{PID_{MU},M_{3},C_{2},V_{MUG}\right\}$ to SD.
**Step 3:**  After receiving the message, SD computes $r_{SD}=B_{1}⊕h(ID_{SD}\left|\right|K_{SD})$, $K_{GSD}=B_{2}⊕h(r_{SD}\left|\right|K_{SD})$, $M_{2}^{*}=M_{3}⊕h(PID_{SD}\left|\right|K_{GSD}\left|\right|r_{SD})$, and $V_{MUG}^{*}=h(PID_{MU}\left|\right|M_{2}^{*}\left|\right|K_{GSD})$. SD checks if $V_{MUG}^{*}\overset{?}{=}V_{MUG}$. If this condition is valid, SD generates a random nonce $RN_{SD}$. Then, SD computes $(h(ID_{MU}||RN_{MU})||h(ID_{G}||RN_{G}))=C_{2}⊕h(K_{GSD}\left|\right|r_{SD})$, $SK=h(h(ID_{MU}||RN_{MU})||h(ID_{G}||RN_{G})||h(ID_{SD}||RN_{SD}))$, $M_{4}=h(PID_{SD}\left|\right|K_{GSD}\left|\right|r_{SD})⊕h(ID_{SD}||RN_{SD})$, and $V_{SD}=h(PID_{MU}||PID_{SD}\left|\right|M_{2}^{*}\left|\right|$$h(ID_{SD}||RN_{SD})||K_{GSD})$. Finally, SD sends $\left\{M_{4},V_{SD}\right\}$ to HGW.
**Step 4:**  Upon receiving the message, HGW computes $h(ID_{SD}||RN_{SD})=M_{4}⊕h(PID_{SD}\left|\right|K_{GSD}\left|\right|r_{SD})$ and $V_{SD}^{*}=h(PID_{MU}||PID_{SD}\left|\right|M_{2}||h(ID_{SD}||RN_{SD})||K_{GSD})$. HGW checks if $V_{SD}^{*}\overset{?}{=}V_{SD}$. Then, HGW computes $SK=h(h(ID_{MU}||RN_{MU})||h(ID_{G}||RN_{G})||ID_{SD}||RN_{SD}))$, $PID_{MU}^{new}=h(PID_{MU}||RN_{MU})$, and $RID_{MU}^{new}=h(PID_{MU}^{new}\left|\right|K_{MUG})$, and computes $M_{5}=h(RID_{MU}||RN_{MU})⊕(h(ID_{G}\left|\right|$$RN_{G})||h(ID_{SD}||RN_{SD})||PID_{MU}^{new})$ and $V_{GSD}=h(PID_{MU}||RN_{MU}||h(ID_{G}||RN_{G})||h(ID_{SD}||RN_{SD})||PID_{MU}^{new}\left|\right|K_{MUG})$.
  HGW stores $\left\{PID_{MU},RID_{MU}\right\}$ with $\left\{PID_{MU}^{new},RID_{MU}^{new}\right\}$ in HGW’s database. Finally, HGW sends $\left\{M_{5},V_{GSD}\right\}$ to MU.
**Step 5:**  After receiving the message, MU computes $PID_{MU}^{new}=h(PID_{MU}\left|\right|RN_{MU})$, $(h(ID_{G}\left|\right|$$RN_{G})||h(ID_{SD}||RN_{SD})||PID_{MU}^{new})=M_{5}⊕h(RID_{MU}||RN_{MU})$ and $V_{GSD}^{*}=h(PID_{MU}$$||RN_{MU}||h(ID_{G}||RN_{G})||h(ID_{SD}||RN_{SD})||PID_{MU}^{new}\left|\right|K_{MUG})$. MU checks if $V_{GSD}^{*}\overset{?}{=}V_{GSD}$. After that, MU computes $SK=h(h(ID_{MU}||RN_{MU})||h(ID_{G}||RN_{G})||h(ID_{SD}\left|\right|$$RN_{SD}))$. Then, MU updates $RID_{MU}^{new}=h(PID_{MU}^{new}\left|\right|K_{MUG})$, $A_{3}^{new}=RID_{MU}^{new}$$⊕h(r_{MU}||$$HPW_{MU})$, and $A_{4}^{new}=K_{MUG}⊕h(RID_{MU}^{new}||HPW_{MU})$. Then, MU replaces $\left\{A_{3},A_{4},PID_{MU}\right\}$ to $\left\{A_{3}^{new},A_{4}^{new},PID_{MU}^{new}\right\}$ in the mobile device. MU computes $M_{6}=h(SK||$$PID_{MU}^{new})$ and sends $M_{6}$ to HGW.
**Step 6:**  After receiving the message from MU, HGW computes $M_{6}^{*}=h(SK||PID_{MU}^{new})$ and checks if $M_{6}^{*}\overset{?}{=}M_{6}$. If it is correct, HGW deletes {$PID_{MU},RID_{MU}$} in the database.

### 6.4. Password Update Phase

**Figure 5:**
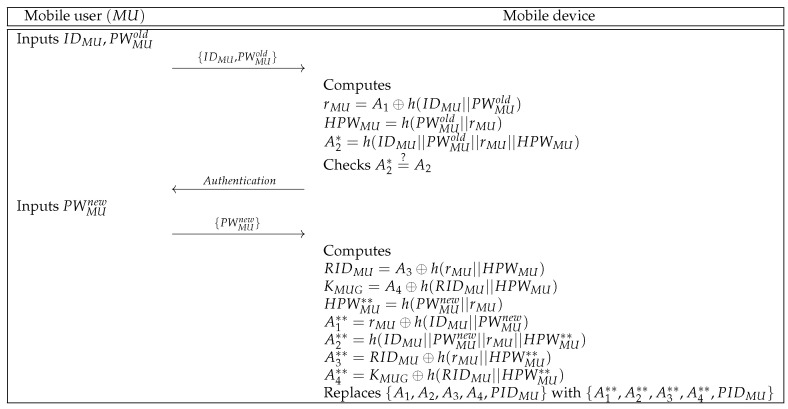
Password update phase of the proposed protocol.

MU can update the password individually. In Figure 5, we represent the password update phase and the detailed steps are below.

**Step 1:**  MU inputs identity and old password $\left\{ID_{MU},PW_{MU}^{old}\right\}$ to the mobile device over a secure channel.
**Step 2:**  Mobile device computes $r_{MU}=A_{1}⊕h(ID_{MU}||PW_{MU}^{old})$, $HPW_{MU}=h(PW_{MU}^{old}\left|\right|r_{MU})$, and $A_{2}^{*}=h(ID_{MU}||PW_{MU}^{old}\left|\right|r_{MU}||HPW_{MU})$. Then, the mobile device checks whether $A_{2}^{*}\overset{?}{=}A_{2}$. If this condition is met, the mobile device sends the authentication message to MU.
**Step 3:**  Upon receiving the authentication message, MU inputs the new password $PW_{MU}^{new}$ to the mobile device.
**Step 4:**  After getting the new password, the mobile device computes $RID_{MU}=A_{3}⊕h(r_{MU}||HPW_{MU})$, $K_{MUG}=A_{4}⊕h(RID_{MU}||HPW_{MU})$, $HPW_{MU}^{**}=h(PW_{MU}^{new}\left|\right|r_{MU})$, $A_{1}^{**}=r_{MU}⊕h(ID_{MU}||PW_{MU}^{new})$, $A_{2}^{**}=h(ID_{MU}||PW_{MU}^{new}\left|\right|r_{MU}||HPW_{MU}^{**})$, $A_{3}^{**}=RID_{MU}⊕h(r_{MU}||HPW_{MU}^{**})$, and $A_{4}^{**}=K_{MUG}⊕h(RID_{MU}||HPW_{MU}^{**})$. Finally, the mobile device replaces $\left\{A_{1},A_{2},A_{3},A_{4},PID_{MU}\right\}$ with $\left\{A_{1}^{**},A_{2}^{**},A_{3}^{**},A_{4}^{**},PID_{MU}\right\}$.

## 7. Security Analysis

This section shows informal and formal security analyses of our protocol using BAN logic, the ROR model, and the AVISPA tool. Through theses analyses, we demonstrate that the proposed protocol prevents various kinds of known attacks.

### 7.1. Informal Security Analysis

We performed informal analysis to describe how our protocol withstands various attacks and supports perfect forward secrecy and mutual authentication.

#### 7.1.1. Mobile User Impersonation Attack

According to Section 1.2, an adversary A can have the lost/stolen mobile device of a legal user MU, and extract secret credentials $\left\{A_{1},A_{2},A_{3},A_{4},PID_{MU}\right\}$ using the power analysis [18,19]. With these values, A can try to impersonate MU by intercepting transmitted messages through an insecure channel. However, A cannot send a valid authentication request message $\left\{M_{1},C_{1},V_{MU}\right\}$ because A cannot calculate $\left\{HPW_{MU},RID_{MU},K_{MUG}\right\}$ without the knowledge of the MU’s real identity $ID_{MU}$, password $PW_{MU}$, and a random nonce $RN_{MU}$. Hence, the proposed protocol resists the mobile user impersonation attack.

#### 7.1.2. Home Gateway Impersonation Attack

Suppose that an adversary A intercepts messages $\left\{PID_{MU},M_{3},C_{2},V_{MUG}\right\}$ and $\left\{M_{5},V_{GSD}\right\}$ over an insecure channel. A can try to calculate the other valid messages $\left\{PID_{MU},M_{3}^{\prime },C_{2}^{\prime },V_{MUG}^{\prime }\right\}$ and $\left\{M_{5}^{\prime },V_{GSD}^{\prime }\right\}$. However, A cannot compute messages, because A has no knowledge of the MU’s real identity $ID_{MU}$ and a random nonce $RN_{MU}$. In addition, A does not know HGW’s real identity $ID_{G}$, a random nonce $RN_{G}$, and the shared secret key $K_{GSD}$. Thus, the proposed protocol withstands the home gateway impersonation attack.

#### 7.1.3. Smart Device Impersonation Attack

An adversary A can try to impersonate SD using the exchanged message $\left\{M_{4},V_{SD}\right\}$. According to Section 1.2, A can extract stored values in the lost/stolen smart device. However, A cannot compute the message because A does not know the SD’s unique identity $ID_{SD}$, secret key $K_{SD}$, and a random nonce $RN_{SD}$. Therefore, our protocol prevents the smart device impersonation attack.

#### 7.1.4. Session Key Disclosure Attack

In accordance with Section 1.2, an adversary A can extract secret credentials $\left\{A_{1},A_{2},A_{3},A_{4},PID_{MU}\right\}$ and $\left\{B_{1},B_{2},PID_{SD}\right\}$ of MU and SD, respectively. To calculate the session key, A should know real identities and random nonces of MU, HGW, and SD. However, A cannot obtain $\left\{ID_{MU},ID_{G},ID_{SD}\right\}$ and $\left\{RN_{MU},RN_{G},RN_{SD}\right\}$ from transmitted messages because these are encrypted with secret keys $\left\{K_{MUG},K_{GSD},K_{SD}\right\}$. Thus, the proposed protocol withstands the session key disclosure attack.

#### 7.1.5. Replay and MITM Attack

We assume that an adversary A intercepts and resends the previous authentication request message $\left\{PID_{MU},M_{1},C_{1},V_{MU}\right\}$ to HGW for the purpose of disguising MU. HGW detects $RN_{MU}$ is not fresh by checking the validity of $V_{MU}$. In addition, even if A tries to modify the authentication request message, A cannot modify $\left\{M_{1},C_{1},V_{MU}\right\}$ without the knowledge of the MU’s real identity $ID_{MU}$, password $PW_{MU}$, a random nonce $RN_{MU}$, and shared secret key $K_{MUG}$. In conclusion, our protocol prevents replay and MITM attacks.

#### 7.1.6. Offline Guessing Attack

After extracting the information from the MU’s mobile device, A can obtain $A_{1}=r_{MU}⊕h(ID_{MU}||PW_{MU})$, $A_{2}=h(ID_{MU}||PW_{MU}\left|\right|r_{MU}||HPW_{MU})$, $A_{3}=RID_{MU}⊕h(r_{MU}\left|\right|$$HPW_{MU})$, and $A_{4}=K_{MUG}⊕h(RID_{MU}||HPW_{MU})$. All of these values are encrypted with $ID_{MU}$ and $PW_{MU}$. If A wants to compromise the security of our protocol, A needs to guess both $ID_{MU}$ and $PW_{MU}$. However, it is a computationally infeasible problem to A according to Section 1.2. As a result, our protocol resists the offline guessing attack.

#### 7.1.7. Stolen Smart Device Attack

Assume that an adversary A obtains SD and extracts secret credentials $\left\{B_{1},B_{2},PID_{SD}\right\}$ stored in the memory through the power analysis attack [20,21]. Although A obtains these values, A cannot get sensitive information of SD because all information stored in the memory is masked with SD’s unique identity $ID_{SD}$ and secret key $K_{SD}$. Thus, the proposed protocol withstands the stolen smart device attack.

#### 7.1.8. Privileged-Insider Attack

In this attack, a privileged-insider adversary A is able to get $PID_{MU}$ during the MU’s registration phase. Then, A can extract secret credentials $\left\{A_{1},A_{2},A_{3},A_{4},PID_{MU}\right\}$ stored in the mobile device. However, since A does not know the MU’s real identity $ID_{MU}$, password $PW_{MU}$, and a random number $r_{MU}$, A cannot calculate the session key $SK=h(h(ID_{MU}||RN_{MU})||h(ID_{G}||RN_{G})||h(ID_{SD}||RN_{SD}))$. Hence, our protocol prevents the privileged-insider attack.

#### 7.1.9. Known Session-Secret Temporary Information Attack

An adversary A can obtain session specific random nonces $\left\{RN_{MU},RN_{G},RN_{SD}\right\}$ to conduct the known session-secret temporary information attack under the CK-adversary model. Even if A knows these secrets, A cannot calculate the session key $SK=h(h(ID_{MU}\left|\right|$$RN_{MU})||h(ID_{G}||RN_{G})||h(ID_{SD}||RN_{SD}))$, because SK consists of MU, HGW, and SD’s identities. Thus, our protocol withstands the known session-secret temporary information attack.

#### 7.1.10. Desynchronization Attack

A desynchronization attack is when an adversary A can modify and block the transmitted messages to make MU, HGW, and SD unable to authenticate in the future. Assume that A tries to modify the messages for desynchronizing the next session. However, as mentioned in Section 7.1.5, A cannot modify the exchanged messages because A has no knowledge about MU’s secret credentials. In addition, we assume that A blocks the transmitted messages to disturb the synchronization. HGW calculates $PID_{MU}^{new}$, generates a verification message $\left\{M_{5},V_{GSD}\right\}$ using $PID_{MU}^{new}$, and sends it to MU. HGW stores the $PID_{MU}^{new}$ with $PID_{MU}$, and MU checks $V_{GSD}$. If the $V_{GSD}$ is correct, MU updates $PID_{MU}^{new}$. MU sends the message $M_{6}$ to HGW to describe that authentication is complete. Then, HGW checks the validation of $M_{6}$. If $M_{6}$ is validated, HGW deletes the old $PID_{MU}$ and $RID_{MU}$. Otherwise, HGW stores them. Through these things, MU and HGW always have synchronized values. Consequently, a desynchronization attack is impossible in our protocol.

#### 7.1.11. Perfect Forward Secrecy

We assume that an adversary A knows long-term secret keys $\left\{K_{RA},K_{MUG},K_{GSD},K_{SD}\right\}$. A can try to calculate the session key $SK=h(h(ID_{MU}||RN_{MU})||h(ID_{G}||RN_{G})||h(ID_{SD}||RN_{SD}))$. However, A cannot affect on the confidentiality of past communications because SK is composed of the random nonces $\left\{RN_{MU},RN_{G},RN_{SD}\right\}$ which is generated for each session. Thus, the proposed protocol provides the perfect forward secrecy.

#### 7.1.12. Mutual Authentication

At the authentication and key agreement phase, MU, HGW, and SD check the message validity. MU checks the validity of $V_{GSD}^{*}$, HGW verifies $V_{MU}^{*}\overset{?}{=}V_{MU}$ and $V_{SD}^{*}\overset{?}{=}V_{SD}$, and SD checks whether $V_{MUG}^{*}\overset{?}{=}V_{MUG}$. If the values are correct, each entity authenticates each other. Therefore, our protocol achieves the mutual authentication.

#### 7.1.13. Anonymity and Untraceability

An adversary A can obtain exchanged messages in the authentication and key agreement phase. However, A cannot obtain real identities of MU, HGW, and SD because these are dependent on $\left\{r_{MU},RN_{G},r_{SD}\right\}$. In addition, MU and HGW update $PID_{MU}$ to $PID_{MU}^{new}=h(PID_{MU}||RN_{MU})$ for every session. It makes all messages are dynamic at every session. Consequently, the proposed protocol provides anonymity and untraceability.

### 7.2. BAN Logic

We performed the formal security analysis with BAN logic to evaluate the secure mutual authentication of the proposed protocol [10,40]. We present the notation of BAN logic in Table 2.

#### 7.2.1. Rules

We describe the rules of BAN logic in the following.

- Message meaning rule (MMR):
  $$\frac{W|\equiv W\overset{skey}{⟷}N,W◃{\left\{S\right\}}_{skey}}{W|\equiv N|∼S}$$
- Nonce verification rule (NVR):
  $$\frac{W|\equiv \#(S),W|\equiv N|∼S}{W|\equiv N|\equiv S}$$
- Jurisdiction rule (JR):
  $$\frac{W|\equiv N|\Rightarrow S,W|\equiv N|\equiv S}{W|\equiv S}$$
- Freshness rule (FR):
  $$\frac{W|\equiv \#(S)}{W|\equiv \#(S,T)}$$
- Belief rule (BR):
  $$\frac{W|\equiv (S,T)}{W|\equiv S}$$

#### 7.2.2. Goals

The following are the main goals to demonstrate that our protocol satisfies the secure mutual authentication.

**Goal 1:**  $MU|\equiv (MU\overset{SK}{⟷}SD)$.
**Goal 2:**  $MU|\equiv SD|\equiv (MU\overset{SK}{⟷}SD)$.
**Goal 3:**  $SD|\equiv (MU\overset{SK}{⟷}SD)$.
**Goal 4:**  $SD|\equiv MU|\equiv (MU\overset{SK}{⟷}SD)$.

#### 7.2.3. Assumptions

We assume the following to initiate states of the proposed protocol.

$A_{1}$: $HGW|\equiv (MU\overset{SK}{⟷}HGW)$
$A_{2}$: $HGW|\equiv \#(RN_{MU})$
$A_{3}$: $SD|\equiv (HGW\overset{K_{GSD}}{⟷}SD)$
$A_{4}$: $SD|\equiv \#(RN_{G})$
$A_{5}$: $HGW|\equiv (HGW\overset{K_{GSD}}{⟷}SD)$
$A_{6}$: $HGW|\equiv \#(RN_{SD})$
$A_{7}$: $MU|\equiv (MU\overset{K_{MUG}}{⟷}HGW)$
$A_{8}$: $MU|\equiv \#(RN_{G})$
$A_{9}$: $MU|\equiv HGW|\Rightarrow MU\overset{h(ID_{G}||RN_{G})||h(ID_{SD}||RN_{SD})}{⇌}SD$
$A_{10}$: $SD|\equiv HGW|\Rightarrow (MU\overset{h(ID_{MU}||RN_{MU})||h(ID_{G}||RN_{G})}{⇌}SD)$
$A_{11}$: $MU|\equiv SD|\Rightarrow (MU\overset{SK}{⟷}SD)$
$A_{12}$: $SD|\equiv MU|\Rightarrow (MU\overset{SK}{⟷}SD)$

#### 7.2.4. Idealized Forms

We present ideal forms of our protocol as below.

$M_{1}$: $MU\to HGW:{\left(PID_{MU},RID_{MU},RN_{MU}\right)}_{K_{MUG}}$
$M_{2}$: $HGW\to SD:(PID_{MU},h(ID_{MU}||RN_{MU}),h(ID_{G}||RN_{G}{),PID_{SD},r_{SD})}_{K_{GSD}}$
$M_{3}$: $SD\to HGW:(PID_{MU},PID_{SD},h(ID_{MU}||RN_{MU}),h(ID_{SD}||RN_{SD}{))}_{K_{GSD}}$
$M_{4}$: $HGW\to MU:(RID_{MU},h(ID_{MU}||RN_{MU}),h(ID_{G}||RN_{G}),h(ID_{SD}||RN_{SD}{))}_{K_{MUG}}$

#### 7.2.5. Proof

We conducted the BAN logic test, and detailed steps are described as follows.

**Step 1:**  From $M_{1}$, we can obtain $S_{1}$.
  $$S_{1}:HGW◃{\left(PID_{MU},RID_{MU},RN_{MU}\right)}_{K_{MUG}}$$
**Step 2:**  Using $S_{1}$ and $A_{1}$ with MMR, we can get $S_{2}$.
  $$S_{2}:HGW\left|\equiv MU\right|∼{\left(PID_{MU},RID_{MU},RN_{MU}\right)}_{K_{MUG}}$$
**Step 3:**  $S_{3}$ can obtained using $S_{2}$ and $A_{2}$ with FR.
  $$S_{3}:HGW|\equiv \#{\left(PID_{MU},RID_{MU},RN_{MU}\right)}_{K_{MUG}}$$
**Step 4:**  Using $S_{2}$ and $S_{3}$ with NVR, we can get $S_{4}$.
  $$S_{4}:HGW\left|\equiv MU\right|\equiv {\left(PID_{MU},RID_{MU},RN_{MU}\right)}_{K_{MUG}}$$
**Step 5:**  We can obtain $S_{5}$ from $M_{2}$.
  $$S_{5}:SD◃(PID_{MU},h(ID_{MU}||RN_{MU}),h(ID_{G}||RN_{G}),PID_{SD},r_{SD})$$
**Step 6:**  $S_{6}$ can obtained using $S_{5}$ and $A_{3}$ with MMR.
  $$S_{6}:SD|\equiv HGW|∼(PID_{MU},h(ID_{MU}||RN_{MU}),h(ID_{G}||RN_{G}{),PID_{SD},r_{SD})}_{K_{GSD}}$$
**Step 7:**  Utilizing $S_{6}$ and $A_{4}$ with FR, we can get $S_{7}$.
  $$S_{7}:SD|\equiv \#(PID_{MU},h(ID_{MU}||RN_{MU}),h(ID_{G}||RN_{G}{),PID_{SD},r_{SD})}_{K_{GSD}}$$
**Step 8:**  For obtaining $S_{8}$, we can use $S_{6}$ and $S_{7}$ with NVR.
  $$S_{8}:SD|\equiv HGW|\equiv (PID_{MU},h(ID_{MU}||RN_{MU}),h(ID_{G}||RN_{G}{),PID_{SD},r_{SD})}_{K_{GSD}}$$
**Step 9:**  From $M_{3}$, we can obtain $S_{9}$.
  $$S_{9}:HGW◃(PID_{MU},PID_{SD},h(ID_{MU}||RN_{MU}),h(ID_{SD}||RN_{SD}{))}_{K_{GSD}}$$
**Step 10:**  For getting $S_{10}$, we can utilize $S_{9}$ and $A_{5}$ with MMR.
  $$S_{10}:HGW|\equiv SD|∼(PID_{MU},PID_{SD},h(ID_{MU}||RN_{MU}),h(ID_{SD}||RN_{SD}{))}_{K_{GSD}}$$
**Step 11:**  For obtaining $S_{11}$, we can use $A_{6}$ and $S_{10}$ with FR.
  $$S_{11}:HGW|\equiv \#(PID_{MU},PID_{SD},h(ID_{MU}||RN_{MU}),h(ID_{SD}||RN_{SD}{))}_{K_{GSD}}$$
**Step 12:**  Using $S_{10}$ and $S_{11}$ with NVR, we can get $S_{12}$.
  $$S_{12}:HGW|\equiv SD|\equiv (PID_{MU},PID_{SD},h(ID_{MU}||RN_{MU}),h(ID_{SD}||RN_{SD}{))}_{K_{GSD}}$$
**Step 13:**  We can get $S_{13}$ from $M_{4}$.
  $$S_{13}:MU◃(RID_{MU},h(ID_{MU}||RN_{MU}),h(ID_{G}||RN_{G}),h(ID_{SD}||RN_{SD}{))}_{K_{MUG}}$$
**Step 14:**  $S_{14}$ can obtained using $S_{13}$ and $A_{7}$ with MMR.
  $$MU|\equiv HGW|∼(RID_{MU},h(ID_{MU}||RN_{MU}),h(ID_{G}||RN_{G}),h(ID_{SD}||RN_{SD}{))}_{K_{MUG}}$$
**Step 15:**  $S_{15}$ can obtained using $S_{14}$ and $A_{8}$ with FR.
  $$S_{15}:MU|\equiv \#(RID_{MU},h(ID_{MU}||RN_{MU}),h(ID_{G}||RN_{G}),h(ID_{SD}||RN_{SD}{))}_{K_{MUG}}$$
**Step 16:**  Using $S_{14}$ and $S_{15}$ with NVR, we can get $S_{16}$.
  $$S_{16}:MU|\equiv HGW|\equiv (RID_{MU},h(ID_{MU}||RN_{MU}),h(ID_{G}||RN_{G}),h(ID_{SD}||RN_{SD}{))}_{K_{MUG}}$$
**Step 17:**  Since the session key is $SK=h(h(ID_{MU}||RN_{MU})||h(ID_{G}||RN_{G})||h(ID_{SD}||RN_{SD}))$, we can obtain $S_{17}$ from $S_{12}$, $S_{16}$, and $A_{9}$.
  $$S_{17}:MU\left|\equiv SD\right|\equiv \left(MU\overset{SK}{⟷}SD\right)\;\left(\mathrm{Goal}2\right)$$
**Step 18:**  From $S_{4}$, $S_{8}$, and $A_{10}$, we can get $S_{18}$.
  $$S_{18}:SD\left|\equiv MU\right|\equiv \left(MU\overset{SK}{⟷}SD\right)\;\left(\mathrm{Goal}4\right)$$
**Step 19:**  $S_{19}$ can obtained from $S_{17}$ and $A_{11}$.
  $$S_{19}:MU|\equiv \left(MU\overset{SK}{⟷}SD\right)\;\left(\mathrm{Goal}1\right)$$
**Step 20:**  $S_{20}$ can obtained using $S_{18}$ and $A_{12}$.
  $$S_{20}:SD|\equiv \left(MU\overset{SK}{⟷}SD\right)\;\left(\mathrm{Goal}3\right)$$

Therefore, MU, HGW, and SD can perform the secure mutual authentication in our protocol.

### 7.3. ROR Model

The session key security of the proposed protocol is demonstrated using the ROR model [11]. We interpret the ROR model before proving the session key security of the proposed protocol. In the authentication and key agreement phase of the proposed protocol, we have three participants $P^{t}$, which are mobile user $P_{MU}^{t_{1}}$, home gateway $P_{HGW}^{t_{2}}$, and smart device $P_{SD}^{t_{3}}$. These are instances $t_{1}$, $t_{2}$, and $t_{3}$ for MU, HGW, and SD, respectively. A can eavesdrop, intercept, or modify transmitted messages through an insecure channel. In addition, A can simulate active and passive attacks by executing various queries defined in the ROR model, such as Execute, CorruptMD, Reveal, Send, and Test queries. Detailed instructions of the queries are below.

- $Execute(P_{MU}^{t_{1}},P_{HGW}^{t_{2}},P_{SD}^{t_{3}})$: A performs this query to obtain transmitted messages over a public channel between MU, HGW, and SD.
- $CorruptMD(P_{MU}^{t_{1}})$: This query represents that A can extract sensitive information stored in the mobile device of MU.
- $Reveal(P^{t})$: This query is that A reveals the current session key SK between $P_{MU}^{t_{1}}$ and $P_{SD}^{t_{3}}$. If an adversary A cannot reveal the session key SK between $P_{MU}^{t_{1}}$ and $P_{SD}^{t_{3}}$ using the $Reveal(P^{t})$ query, then SK is secure.
- $Send(P^{t},M)$: With this query, A can send the message *M* to $P^{t}$ and receive a response message.
- $Test(P^{t})$: Before the start of the game, a fair coin fc is tossed and the result becomes only known to A. A uses this result to make a decision of the Test query. If A runs the Test query and the session key SK is fresh, $P^{t}$ returns SK for fc = 1 or a random number for fc = 0. Otherwise, it returns a null (*⊥*).

After A performs the Test query on $P^{t}$, A must distinguish the result value. A uses the output of the Test query for checking the consistency of the random bit fc. A wins the game when the guessed bit $fc^{\prime }$ is equal to fc. Moreover, all participants have access to a collision-resistant cryptographic one-way hash function h(·). We model h(·) as a random oracle, Hash.

#### 7.3.1. Security Proof

We prove the session key security of the proposed protocol using Zipf’s law [41].

**Theorem** **1.** *A can break the session key security of the proposed protocol. We denote the advantage of A running in polynomial time as $Adv_{A}$. Then, we obtain the following.*

$$Adv_{A}\leq \frac{q_{h}^{2}}{\left|Hash\right|}+2\left\{C\cdot q_{send}^{s}\right\}$$

*Here, $q_{h}$ is the number of Hash queries, |Hash| is the range space of the hash function h(·), and $q_{send}$ is the number of Send queries. In addition, C and s denote Zipf’s parameters [41].*

**Proof.**  The proof of Theorem 1 is similar as presented in [42,43]. We prove the session key security through a sequence of four games, $GM_{i}$, where i∈[0,3]. $Succ_{A,i}$ indicates the event that A wins $GM_{i}$ by guessing the random bit fc correctly. We denote the advantage of A winning the game $GM_{i}$ as $Pr[Succ_{A,GM_{i}}]$. In the following, we describe each game.

- $GM_{0}$: This game allows A to execute the real attack against the proposed protocol. A chooses a random bit fc at the beginning of $GM_{0}$. Then, we obtain the following in accordance with this game.
  (1) $$Adv_{A}=\left|2Pr\left[Succ_{A,GM_{0}}\right]-1\right|$$
- $GM_{1}$: In this game, A runs the $Execute(P_{MU}^{t_{1}},P_{HGW}^{t_{2}},P_{SD}^{t_{3}})$ query and eavesdrops transmitted messages $\left\{PID_{MU},M_{1},C_{1},V_{MU}\right\}$, $\left\{PID_{MU},M_{3},C_{2},V_{MUG}\right\}$, $\left\{M_{4},V_{SD}\right\}$, and $\left\{M_{5},V_{GSD}\right\}$. Then, A executes Reveal and Test queries to validate whether the derived session key is real or not. In our protocol, the session key is constructed as $SK=h(h(ID_{MU}||RN_{MU})||h(ID_{G}||RN_{G})||h(ID_{SD}||RN_{SD}))$. To derive the session key, A needs to know the identities and random nonces of MU, HGW, and SD. Consequently, there are no instances in which A increases $GM_{1}$’s winning probability. Therefore, $GM_{0}$ and $GM_{1}$ turn out to be indistinguishable, and we can obtain the following.
  (2) $$Pr\left[Succ_{A,GM_{1}}\right]=Pr\left[Succ_{A,GM_{0}}\right]$$
- $GM_{2}$: To obtain the session key, A performs Hash and Send queries in this game. A can perform an active attack by modifying exchanged messages. However, all exchanged messages are constructed with secret credentials and random nonces, and protected using one-way hash function h(·). In addition, A is difficult to derive secret credentials and random nonces because it is a computationally infeasible problem according to the property of h(·). Hence, we can get the following result through the use of birthday paradox [44].
  (3) $$|Pr\left[Succ_{A,GM_{2}}\right]-Pr\left[Succ_{A,GM_{1}}\right]|\leq \frac{q_{h}^{2}}{2|Hash|}$$
- $GM_{3}$: In the final game $GM_{3}$, A can try to get the session key with the CorruptMD query. By the CorruptMD query, A can extract sensitive values $\left\{A_{1},A_{2},A_{3},A_{4}\right\}$ stored in the mobile device of MU. Sensitive values are expressed as $A_{1}=r_{MU}⊕h(ID_{MU}||PW_{MU})$, $A_{2}=h(ID_{MU}||PW_{MU}\left|\right|r_{MU}||HPW_{MU})$, $A_{3}=RID_{MU}⊕h(PID_{MU}||HPW_{MU})$, and $A_{4}=K_{MUG}⊕h(RID_{MU}||HPW_{MU})$. Since A has no knowledge of $ID_{MU}$ and $PW_{MU}$, A cannot derive secret values $r_{MU}$ and $K_{MUG}$ from the extracted values. Besides, it is a computationally infeasible task for A to guess $ID_{MU}$ and $PW_{MU}$ simultaneously. In conclusion, $GM_{2}$ and $GM_{3}$ are indistinguishable. By utilizing Zipf’s law, the following result can be obtained.
  (4) $$|Pr\left[Succ_{A,GM_{3}}\right]-Pr\left[Succ_{A,GM_{2}}\right]|\leq C\cdot q_{send}^{s}$$
  As all games have been run, A must guess the bit for winning the game. Therefore, we can obtain the following result.
  (5) $$Pr\left[Succ_{A,GM_{3}}\right]=\frac{1}{2}$$
  From Equations (1) and (2), we obtain the result as follows.
  (6) $$\frac{1}{2}Adv_{A}=|Pr\left[Succ_{A,GM_{0}}-\frac{1}{2}\right]|=|Pr\left[Succ_{A,GM_{1}}-\frac{1}{2}\right]|.$$
  With Equations (5) and (6), we derive the below equation.
  (7) $$\frac{1}{2}Adv_{A}=\left|Pr\left[Succ_{A,GM_{1}}\right]-Pr\left[Succ_{A,GM_{3}}\right]\right|.$$
  By using the triangular inequality, we can have the following result with Equations (4), (5), and (7).
  (8) $$\begin{array}{cc} \frac{1}{2}Adv_{A} & =|Pr\left[Succ_{A,GM_{1}}\right]-Pr\left[Succ_{A,GM_{3}}\right]|\\ & \leq |Pr\left[Succ_{A,GM_{1}}\right]-Pr\left[Succ_{A,GM_{2}}\right]|\\ & \;+|Pr\left[Succ_{A,GM_{2}}\right]-Pr\left[Succ_{A,GM_{3}}\right]|\\ & \leq \frac{q_{h}^{2}}{2|Hash|}+C\cdot q_{send}^{s} \end{array}$$
  Finally, by multiplying both sides of Equation (8) by two, we can obtain the required result.
  (9) $$Adv_{A}\leq \frac{q_{h}^{2}}{\left|Hash\right|}+2\left\{C\cdot q_{send}^{s}\right\}$$

Therefore, we prove Theorem 1. □

### 7.4. AVISPA Tool

We utilized the AVISPA tool [7,8,9] to verify the security of our protocol against MITM and replay attacks. The AVISPA tool uses a role based language, High-Level Protocols Specification Language (HLPSL), to specify actions of each protocol participant [45]. For the security analysis, the HLPSL is entered and translated into the Intermediate Format (IF) in the AVISPA tool. If the IF becomes the input of the back-end, the back-end outputs the security analysis result as the Output Format (OF). The back-end of the AVISPA tool consists of four components, including SAT-based Model-Checker (SATMC), Tree-Automata-based Protocol Analyzer (TA4SP), On-the-Fly-Model-Checker (OFMC), and CL-based Attack Searcher (CL-AtSe). If the OF is SAFE for the back-end, the proposed protocol prevents MITM and replay attacks. We use OFMC and CL-AtSe for the proposed protocol, since SATMC and TA4SP do not support XOR operations.

#### 7.4.1. Specifications of the Proposed Protocol

**Figure 6:**
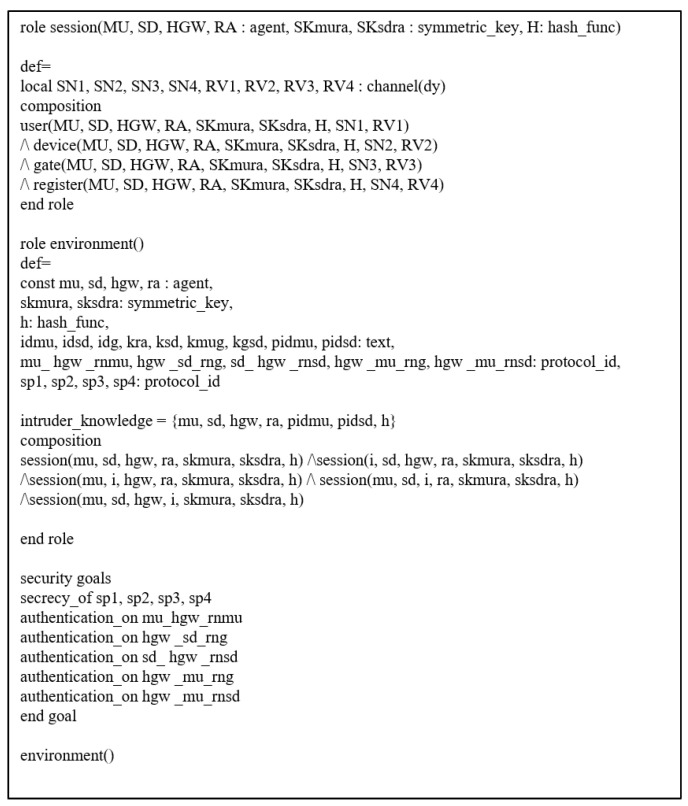
Roles of session, environment, and security goals.

We set up the session, environment, and security goals using the HLPSL language. Details of these are shown in Figure 6. In session and environment, we specify instances of each role and construct the whole protocol session. In addition, we state the security goals of the proposed protocol. secrecy is used to check secret values are explicitly undisclosed and authentication is used to verify the validity of secret values between entities. Through secrecy and authentication, we can confirm that the proposed protocol is resistant to MITM and replay attacks.

**Figure 7:**
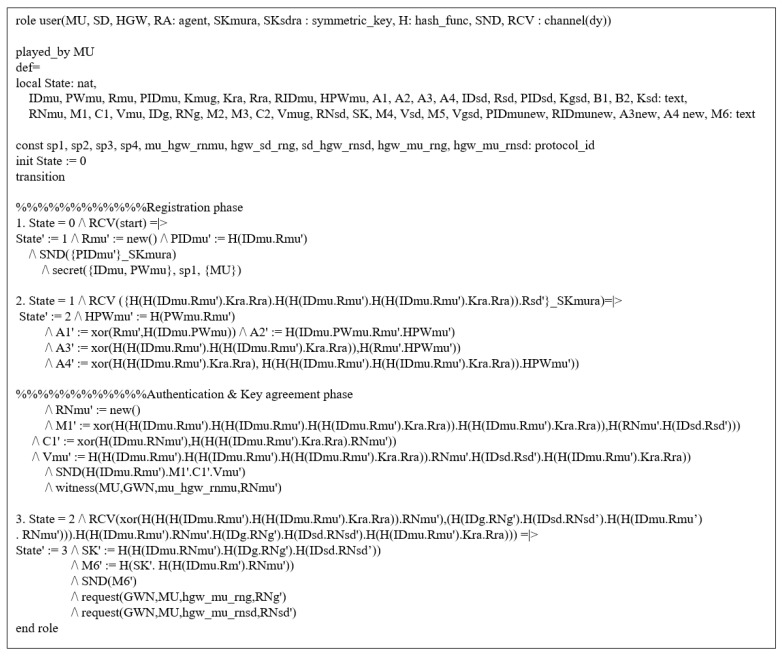
Roles of MU.

As shown in Figure 7, if the registration process is started at state 0, MU generates identity $ID_{MU}$ and password $PW_{MU}$, and calculates $PID_{MU}$ at state 1. Then, MU sends the registration request message $\left\{PID_{MU}\right\}$ to RA. After receiving secret values $\left\{K_{MUG},RID_{MU}\right\}$ from RA, MU updates the state from 1 to 2. Then, MU stores secret values encrypted with the $ID_{MU}$ and $PW_{MU}$ in the mobile device. Then, MU transmits the authentication request message $\left\{PID_{MU},M_{1},C_{1},V_{MU}\right\}$ to HGW. Upon receiving the message $\left\{M_{5},V_{GSD}\right\}$ in state 2, MU updates the state from 2 to 3 and checks $V_{GSD}^{*}\overset{?}{=}V_{GSD}$. If the condition is met, MU authenticates HGW successfully. Then, MU computes $M_{6}$ and sends it to HGW. The roles of HGW, SD, and RA are similar to the roles of MU.

#### 7.4.2. Result of AVISPA

**Figure 8:**
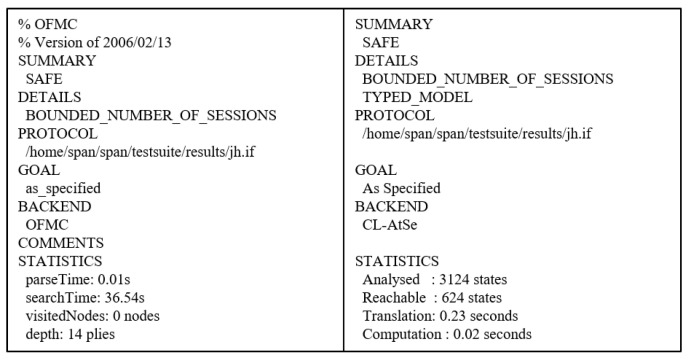
Results of analysis using OFMC and CL-AtSe.

We use OFMC and CL-AtSe for XOR operations to show the security analysis result. The OFMC estimates that the proposed protocol withstands the MITM attack, and CL-AtSe assesses our protocol is resistant to the replay attack. Figure 8 shows the OF of OFMC and CL-AtSe back-ends for the proposed protocol. The output shows that the proposed protocol is SAFE in OFMC and CL-AtSe back-ends. Thus, our protocol successfully satisfies the specified security goals. In other words, our protocol withstands MITM and replay attacks.

## 8. Performance and Security Analyses

This section shows the comparison results of the proposed protocol with similar protocols [6,31,34,38], including computational and communication costs, and security properties.

### 8.1. Computational Costs

The computational costs are analyzed for our protocol and related existing protocols [6,31,34,38]. For comparison, we refer to the work [46]. $T_{m}$, $T_{R}$, $T_{h}$, and $T_{s}$ denote the execution times of an ECC point multiplication (≈7.3529 ms), fuzzy extractor function (≈7.3529 ms), a hash function (≈0.0004 ms), and symmetric key encryption/decryption (≈0.1303 ms), respectively. Table 3 contains the result of the computational costs comparison. Although the proposed protocol has a slightly higher computational cost than the low-security-risk path of Xiang and Zheng’s protocol [6], our protocol provides more robust security. Moreover, the proposed protocol has a lower computational cost compared with the other related protocols, except for the low-security-risk path of Xiang and Zheng’s protocol [6].

### 8.2. Communication Costs

The communication cost of our protocol is compared to those costs of other related protocols [6,31,34,38]. Referring to the paper [31], we define that an ECC point, symmetric key encryption/decryption, hash function, random number, identity, and timestamp are 320, 256, 160, 160, 128, and 32 bits. We estimate the message header as Internet Protocol version 4 (IPv4) packet header, 4 bits. In the authentication and key agreement phase of the proposed protocol, exchanged messages $\left\{PID_{MU},M_{1},C_{1},V_{MU}\right\}$, $\left\{PID_{MU},M_{3},C_{2},V_{MUG}\right\}$, $\left\{M_{4},V_{SD}\right\}$, $\left\{M_{5},V_{GSD}\right\}$, and $M_{6}$ need 640, 640, 320, 20, and 160 bits, respectively. Consequently, our protocol has 2080 bits as the total communication cost. In Table 4, we show the results of the communication costs comparison. Although our protocol has a higher communication cost than some of the existing protocols [6,31,38], it provides more efficient computational costs and security.

### 8.3. Security Properties

In Table 5, we present security properties of the proposed protocol and those of models by Shuai et al. [31], Wazid et al. [34], Banerjee et al. [38], and Xiang and Zheng [6]. In contrast with the other protocols [6,31,34,38], our protocol prevents more attacks. Thus, the proposed protocol meets more security requirements compared to related protocols.

## 9. Conclusions

We proved that Xiang and Zheng’s protocol does not perform secure mutual authentication. We also discovered that their protocol is vulnerable to impersonation, stolen smart device, and session key disclosure attacks. To deal with the security threats to Xiang and Zheng’s protocol, we proposed a secure and lightweight authentication protocol for IoT-based smart homes. We demonstrated that the proposed protocol is secure against various attacks, including impersonation, replay, MITM, and session key disclosure attacks. We performed the BAN logic test to show that our protocol ensures secure mutual authentication. Furthermore, we demonstrated that the proposed protocol provides session key security and resists replay and MITM attacks by utilizing the ROR model and the AVISPA tool. We compared our protocol with associated existing protocols in terms of security properties, and computational and communication costs. In conclusion, our protocol provides better security and low computational costs. When we consider all perspectives of security and costs, our protocol is suitable for practical IoT-based smart home environments. In the future, we will develop a better protocol and implement it in an actual environment.

## Figures and Tables

**Table 1:** Notation.

**Notation**	**Description**
MU	Mobile user
HGW	Home gateway
SD	Smart device
RA	Registration authority
$ID_{MU}$	Identity of MU
$ID_{G}$	Identity of HGW
$ID_{SD}$	Identity of SD
$PID_{MU}$	Pseudo identity of MU
$PID_{SD}$	Pseudo identity of SD
$PW_{MU}$	Password of MU
$K_{RA}$	Master key of RA
$K_{SD}$	Secret key of SD
$K_{MUG}$	Shared secret key between MU and HGW
$K_{GSD}$	Shared secret key between HGW and SD
$r_{MU}$, $r_{RA}$, $r_{SD}$, $RN_{MU}$, $RN_{G}$, $RN_{SD}$	Random number
SK	Session key between MU and SD
h(·)	One-way hash function
$E_{K}\left(\cdot \right)/D_{K}\left(\cdot \right)$	Symmetric encryption/decryption using key *K*
⊕	XOR operation
||	Concatenation operation
*T*	Timestamp
ΔT	Maximum transmission delay
$HE_{i,L}/HE_{i,H}$	Message header at the low/low security risk

**Table 2:** BAN logic notation.

**Notation**	**Description**
skey	Secret key
W|≡S	*W***believes** statement *S*
#S	Statement *S* is **fresh**
W◃S	*W***receives** statement *S*
W|∼S	*W* once **said** *S*
W⇒S	*W***controls** statement *S*
$<S{>}_{T}$	Statement *S* is **combined** with secret statement *T*
${\left\{S\right\}}_{skey}$	Statement *S* is **masked** by skey
$W\overset{skey}{⟷}N$	*W* and *N* **share** skey to communicate with each other
$W\overset{skey}{⇌}N$	skey is known only to *W*, *N*, and trusted principals of *W* and *N*

**Table 3:** Computational costs comparison.

**Protocol**	**Total**	**Computational Costs**
Shuai et al. [31]	$3T_{m}+16T_{h}$	22.0651 ms
Wazid et al. [34]	$25T_{h}+1T_{R}+4T_{s}$	7.8841 ms
Banerjee et al. [38]	$26T_{h}+1T_{R}$	7.3633 ms
Xiang and Zheng [6]	Low-security risk: $11T_{h}$	0.0044 ms
	High-security risk: $11T_{h}+4T_{s}$	0.5256 ms
Ours	$42T_{h}$	0.0168 ms

**Table 4:** Communication costs comparison.

**Protocol**	**Communication Costs**	**Number of Messages**
Shuai et al. [31]	(960 + 320 + 320 + 320) = 1920 bits	4
Wazid et al. [34]	(480 + 960 + 512 + 1408) = 3360 bits	4
Banerjee et al. [38]	(448 + 320 + 320 + 320) = 1408 bits	4
Xiang and Zheng [6]	Low-security risk: (132 + 324 + 324) = 780 bits	3
	High-security risk: (132 + 676 + 676) = 1484 bits	3
Ours	(640 + 640 + 320 + 320 + 160) = 2080 bits	5

**Table 5:** Security properties.

Security Properties	[31]	[34]	[38]	[6]	Ours
Impersonation attack	∘	∘	∘	×	∘
Session key disclosure attack	∘	∘	∘	×	∘
Replay attack	∘	∘	∘	∘	∘
MITM attack	∘	∘	∘	∘	∘
Off-line guessing attack	×	∘	∘	∘	∘
Stolen smart device attack	-	-	-	×	∘
Privileged-insider attack	∘	∘	∘	×	∘
Known session-secret temporary information attack	-	-	∘	×	∘
Desynchronization attack	∘	×	-	×	∘
Perfect forward secrecy	×	×	-	×	∘
Mutual authentication	∘	∘	∘	×	∘
Anonymity	∘	×	×	×	∘
Untraceability	∘	∘	×	×	∘

∘: Secure. ×: Insecure. -: Not considered.

## Data Availability

Not applicable.
